# Ecosystem Overfishing in the Ocean

**DOI:** 10.1371/journal.pone.0003881

**Published:** 2008-12-10

**Authors:** Marta Coll, Simone Libralato, Sergi Tudela, Isabel Palomera, Fabio Pranovi

**Affiliations:** 1 Institut de Ciènces del Mar, ICM-CSIC, Passeig Marítim de la Barceloneta, Barcelona, Spain; 2 Department of Biology, Dalhousie University, Halifax, Nova Scotia, Canada; 3 Istituto Nazionale di Oceanografia e di Geofisica Sperimentale–OGS, Sgonico-Zgonik, Italy; 4 World Wide Fund for Nature, WWF, Mediterranean Programme Office, Barcelona, Spain; 5 Dipartimento di Scienze Ambientali, Università Ca' Foscari, Venezia, Italy; University of Zurich, Switzerland

## Abstract

Fisheries catches represent a net export of mass and energy that can no longer be used by trophic levels higher than those fished. Thus, exploitation implies a depletion of secondary production of higher trophic levels (here the production of mass and energy by herbivores and carnivores in the ecosystem) due to the removal of prey. The depletion of secondary production due to the export of biomass and energy through catches was recently formulated as a proxy for evaluating the ecosystem impacts of fishing–i.e., the level of ecosystem overfishing. Here we evaluate the historical and current risk of ecosystem overfishing at a global scale by quantifying the depletion of secondary production using the best available fisheries and ecological data (i.e., catch and primary production). Our results highlight an increasing trend in the number of unsustainable fisheries (i.e., an increase in the risk of ecosystem overfishing) from the 1950s to the 2000s, and illustrate the worldwide geographic expansion of overfishing. These results enable to assess when and where fishing became unsustainable at the ecosystem level. At present, total catch per capita from Large Marine Ecosystems is at least twice the value estimated to ensure fishing at moderate sustainable levels.

## Introduction

Fisheries can cause depletion of target and non-target species [1], [2], trigger indirect effects in marine populations and communities [3], [4], and modify the structure and function of marine ecosystems [5], [6]. Complex and sometimes synergistic effects of exploitation and environmental variability that propagate through the entire trophic web are frequent causes of failure in fisheries management [7]. Thus, current strategies need to incorporate ecosystem, together with population and community-level assessments to evaluate the sustainable exploitation of marine ecosystems.

Several efforts to quantify the pressure exerted by fishing activities on marine ecosystems worldwide have led to the development of ecological indices. Three important ones are (i) the Primary Production Required to sustain marine catches (PPR, 8), normalized to the primary production (%PPR) and representing catches of different species in uniform energetic units; (ii) the mean Trophic Level of catches (TL_c_, 5), which allows for the evaluation of the trophic position of marine organisms removed from exploited ecosystems, and showed a global trend of fishing down marine food webs; and (iii) the Fishing in Balance index (FiB, 9), which integrates the TL of caught species and the Transfer Efficiency (TE) of energy flows in the food web, and it allows to evaluate if exploitations at different trophic levels are ecologically balanced over time. In order to obtain a comprehensive measure to quantify the consequences of marine fishing activities at an ecosystem level, the total losses in secondary production, due to fishing was recently formulated integrating previous analyses [5], [8], [11] and proposed as a proxy for evaluating the ecosystem impacts of fishing [10]. Secondary production is here the production of mass and energy by all consumers in the ecosystem, thus including both herbivores and carnivores.

Fisheries catches represent a net export of mass and energy that can no longer be used by trophic levels higher than those fished. Thus, exploitation implies a depletion of secondary production of higher trophic levels due to the removal of prey. Based on this assumption, a new method was developed to quantify the loss in secondary production (L index) due to the removal of marine organisms through catches (expressed as PPR equivalents) compared to a theoretical unfished situation [10, see materials and methods]. Reference levels for the L index were quantified by using a set of well documented mass balance models representative of exploited ecosystems distributed worldwide and considering model-independent information on ecosystem status [10]. Each model was classified as representing an overfished or sustainably fished ecosystem based on the ecosystem overfishing definition *sensu* Murawski [12]. Using the frequency distribution of L values it was possible to calculate, for each ecosystem, the probability of the ecosystem to be sustainably fished (p_sust_). These estimates allowed deriving a relationship between L and p_sust_ that can be used to evaluate the risk of ecosystem overfishing [10, see materials and methods]. Ecosystem-based Maximum Sustainable Catches (EMSC) can be then estimated by setting p_sust_ at for e.g., 75% (EMSC_75_) and 95% (EMSC_95_) and assuming constant fishing strategies, i.e. by maintaining current TL_c_ [10]–[11].

In order to quantify L at a global scale and evaluate the global sustainability of marine fisheries we use here the best available geographically referenced database of world catches [the *Sea Around Us Project* SAUP database, 13] that provides detailed catch data allowing for a reliable estimation of PPR and TL_c_, and contains P_1_ estimates. These, together with frequency distribution of TE for ecosystem types [10], allowed us to estimate density functions for L and p_sust_ for 66 Large Marine Ecosystems (LMEs), and 17 FAO areas (open sea oceanic areas outside LMEs). The L index and p_sust_ were computed for each area and for each year of the period 1950 to 2004 using both official catches, and catches corrected to include discards [14] and different estimates of Illegal, Unreported or Unregulated catches [IUU], [ 15]–[17].

## Results and Discussion

### Loss in production and risk of overfishing in the ocean

**Figure 1:**
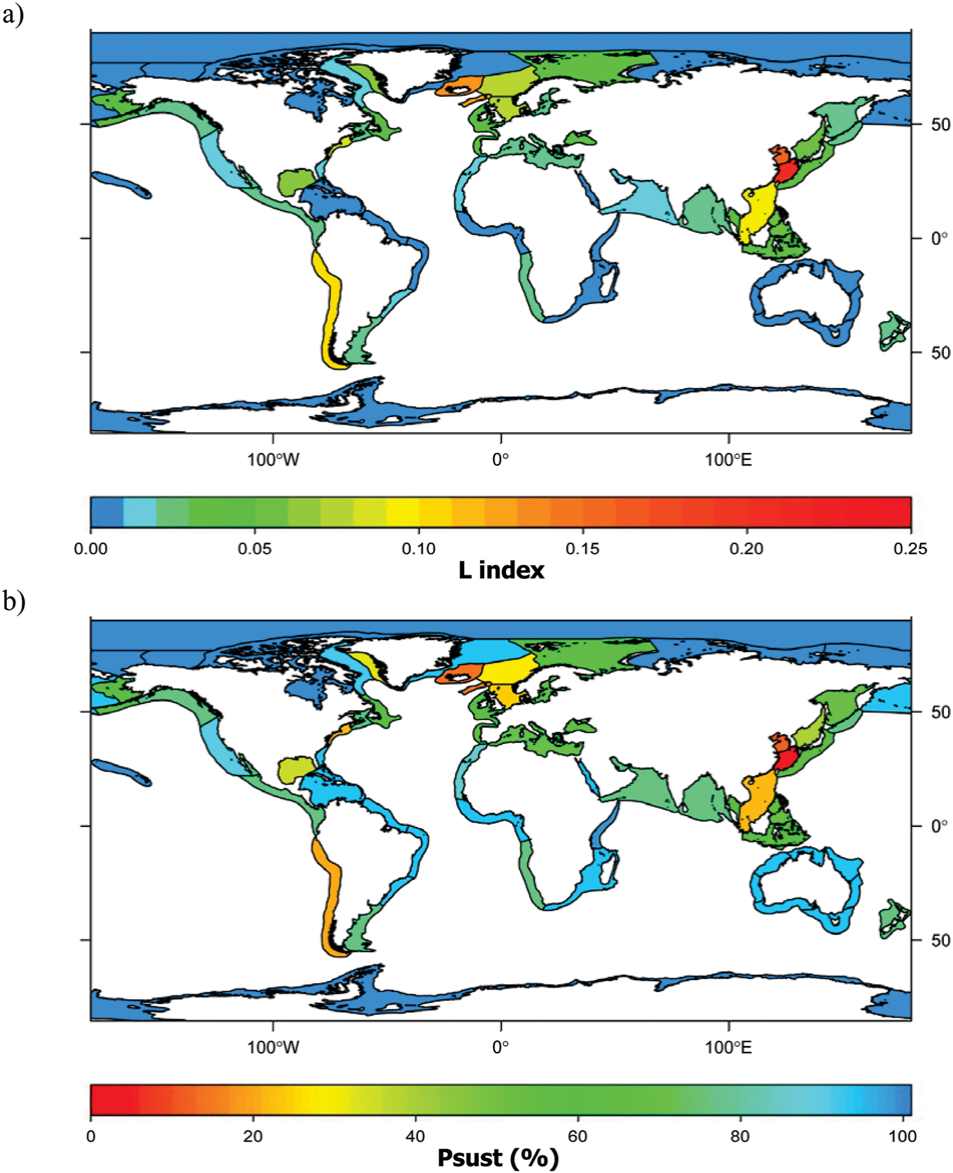
Ecosystem overfishing assessment for Large Marine Ecosystems during 2000–2004: a. Loss in production index (L) (values in the range 0–0.25), and b. Probability of being sustainably fished (p_sust_, %), both taking into account official catches, discards and unreported estimates of 30% (sources: 13–17).

Results accounting for discards and IUU assumed as 30% of official landings indicate that from 2000 to 2004 several LME areas suffered high losses in secondary production due to fishing (Figure 1a) and had fisheries with low sustainability levels (p_sust_≪75%) (Figure 1b; see Table S1 in supplementary material for detailed information on LMEs and open sea FAO areas). This was especially evident for systems located in East Asia, Northern Europe, North Atlantic and the Pacific coast of South America. For temperate and high latitude LMEs, the systems with lowest sustainability estimates were the Sea of Japan (p_sust_ = 40.9%), West Greenland Shelf (33.2%), Norwegian Shelf (27.4%), North Sea (24.2%) and North-eastern US Continental Shelf (22.9%), Faroe Plateau (15.1%), Iceland Shelf (14.3%), and Yellow Sea (12.9%). Within tropical LMEs, the Sulu-Celebes Sea (37.9%) and the Gulf of Mexico (35.4%) had the lowest p_sust_ values. Seas around China showed particularly intense exploitation (East China Sea p_sust_ = 0%, South China Sea p_sust_ = 22.8%), despite the SAUP database has been corrected for over-reporting of catches in the area [18]. For upwelling areas, the Humboldt Current registered the lowest sustainability (20.5%). Conversely, high sustainability was identified for fisheries in high latitude areas of the Arctic and Antarctica, as well as for Australia, Eastern Africa and North-eastern South America. Several open sea areas (FAO areas) also showed high levels of sustainability (p_sust_≥95%), with the Western-Central Pacific ocean (p_sust_ = 63.7%) scoring the lowest. When aggregating data on the basis of ecosystem type (Table 1), upwelling LMEs registered the lowest mean values of fisheries' sustainability (p_sust_ = 53.8%). This is chiefly due to the high catch rates of lower trophic organisms, mainly small pelagic fish (e.g., sardines and anchovies). Temperate and high latitude, and tropical areas, ranked intermediate with mean p_sust_ values of 63.7% and 71.60%, respectively. All FAO areas combined showed the highest mean probability of being sustainably fished (p_sust_ = 95.7%). However, higher risk of overfishing was assessed when higher percentages of IUU were considered (Table 1 and Table S1 supplementary material). Open sea areas, for example, showed decreasing p_sust_ to 94.4%, 63.7%, 53.7% and 20.5% when IUU% increased by 100%, 300%, 500% and 1000% from the initial 30% IUU adopted (Table S2 supplementary material).

**Table 1:** Global assessment of ecosystem overfishing for Large Marine Ecosystem (LME) types and the open sea (FAO areas) during 2000–2004.

Region/area	Temperate & high latitude LME	Tropical LME	Upwelling LME	Open Sea (FAO areas)
Surface (km^2^)	42715207	31567951	10134019	216557627
TE (median)	13.7	9.7	4	11.8
P1	193.27	233.85	323.98	111.92
TL_c_	3.34	3.28	2.94	3.72
Total catch	36103.2	25559.6	20501.1	14470.5
Scenario^1*^				
PPR(%)	5.89	9.21	55.62	3.07
Lindex	0.028	0.019	0.039	0.004
Psust(%)	70.05	77.09	63.65	95.92
Scenario^2*^				
PPR(%)	8.13	12.71	76.76	4.24
Lindex	0.038	0.026	0.054	0.006
Psust(%)	63.65	71.6	53.75	95.7
Scenario^3*^				
PPR(%)	9.75	15.25	92.11	7.93
Lindex	0.046	0.032	0.065	0.011
Psust(%)	59.59	63.68	33.18	94.38
***EMSC***				
EMSC_75_	19189.7	19638.9	7741.7	605908.1
error (+/−)	12366.5	12656.0	4989.0	390468.9
EMSC_95_	6911.0	7072.8	2788.1	218211.9
error (+/−)	6911.0	6588.7	2597.2	203276.0

Calculations take into account official catch data and official data corrected with discards and unreported catches (sources: 13–17, and additional simulations). EMSC values for individual LMEs and the open sea (FAO areas) in 2004 have been pooled together to yield global EMSC on a marine biome basis. LME: Large Marine Ecosystems (n = 66); FAO areas: open sea areas excluding LMEs (n = 17); TE: Transfer efficiency (median); P1: mean primary production (gC·m^−2^·yr^−1^); TLc: mean trophic level of the catch; Total catch for 2004 assuming landings with discards and 30% IUU estimates (10^3^·yr^−1^). PPR(%): mean primary production required to sustain the catch relative to primary production; L index: mean Loss in production index; Psust(%): mean probability of being sustainably fished. EMSC: Ecosystem-based Maximum Sustainable Catches with psust = 75% (EMSC75) and psust = 95% (EMSC95) in 2004 (10^3^·yr^−1^). (^*^) Scenarios include official catch (1), landings with discards and 30% IUU estimates (2), landings with discards and 50% IUU estimates for LME and 100% IUU for FAO open sea areas (3).

Results from LMEs are consistent with published regional case studies [e.g. 1]–[2], [19]. Moreover, although discards and IUU catches were not included in a recent global assessment of cumulative human impacts in marine ecosystems [20], results of this assessment are in general agreement with ours. Namely, Northern Europe, the North Atlantic, and East Asia showed the highest predicted cumulative human impacts whereas the high-latitude areas showed the lowest. Discrepancies between the two assessments were observed for areas such as the Gulf of Mexico, the Pacific-American coast, and upwelling zones where effects of fishing represent the most important human impact [1], [16], [21]–[22] compared to those of other anthropogenic activities.

### Historical risk of ecosystem overfishing

**Figure 2:**
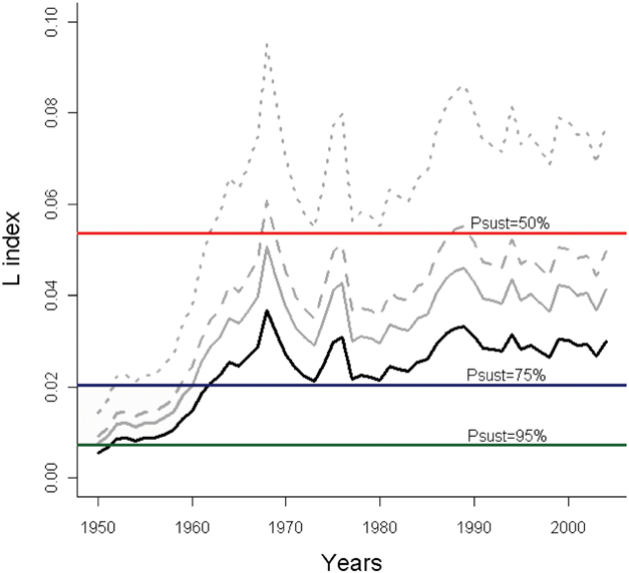
Assessment through time (1950–2004) for Loss in production (L index) with reference levels of 50%, 75% and 95% probability of being sustainably fished. Official catch data (black) and corrected catch data with simulations of discards and unreported catches (grey scale; 30% IUU: solid line, 50% IUU: dashed line, 100% IUU: dotted line) are shown.

**Figure 3:**
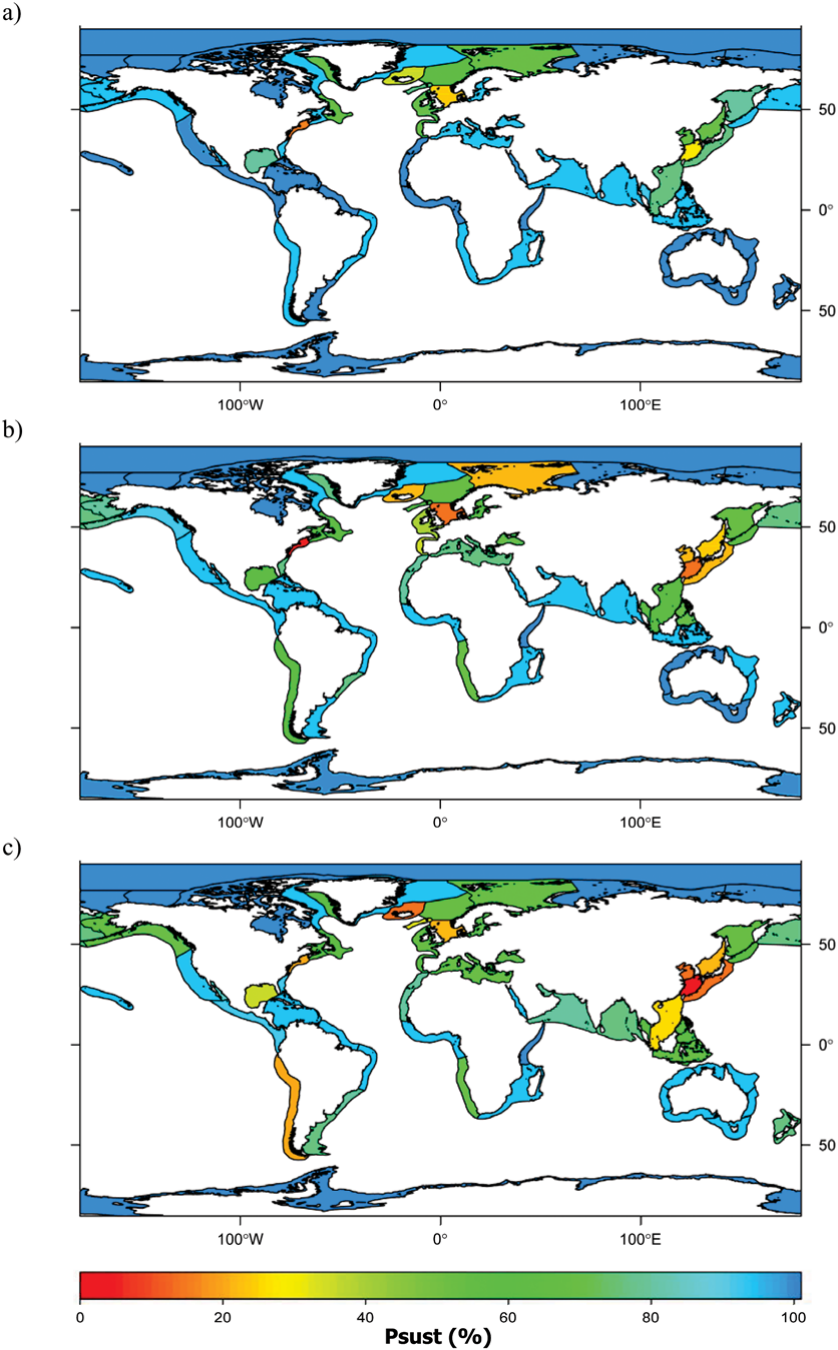
Historical ecosystem overfishing assessment for Large Marine Ecosystems: probability (%) of being sustainably fished (p_sust_, %) during the a. 1950s, b. 1970s, and c. 1990s, taking into account official catches, discards and 30% unreported estimates (sources: 13–17).

Analyses of the global sustainability of exploitations from 1950 to 2004 demonstrate higher sustainable levels of fishing activities during the 1950s (Figure 2). However, the spatial dynamics of exploitation indicate that signs of ecosystem overfishing were already detectable in various LMEs of Northern Europe, the North Atlantic, East Asia, and the Gulf of Mexico during the 1950s (Figure 3a). During the 1960s, L registered a notable increase as the result of vast expansions in global fishing effort at the end of the 1950s [16]. This is especially evident for the Humboldt and Benguela areas (Figure 3b), coinciding with the first collapses of fisheries targeting small pelagic fishes [21]–[22]. The 1970s showed a stabilization of global catches (Figure 2) reflected in a more sustainable phase. Successively, L increased again in the 1980s with new reports of fisheries' collapses [e.g., groundfish in the North Atlantic, 23]. From the 1990s until 2004, L can generally be described as having reached a plateau (Figure 2), although lower sustainabilities have been recorded in areas such as the Arabian Sea, the Bay of Bengal, the Indonesian and South China Seas, New Zealand, and the Norwegian Shelf (Figure 3c, Figure 1b). Slight increases in sustainability have been observed in areas such as the North Sea, the North-eastern US shelf, and the Scotian Shelf due to improvements in fisheries management practices. However, the risk of overexploitation in these areas remains high (see Tables S1, S3, S4, S5).

Overall, these results are consistent with the general expansion over time of fisheries from higher to lower trophic level organisms, from coastal to deeper areas, from higher to lower latitudes, and with the development of more efficient fishing methods [8], [16], [24]. Lower sustainabilities were earlier achieved if higher percentages of IUU catches were included (Figure 2).

### Estimates of Ecosystem-based Maximum Sustainable Catches

**Figure 4:**
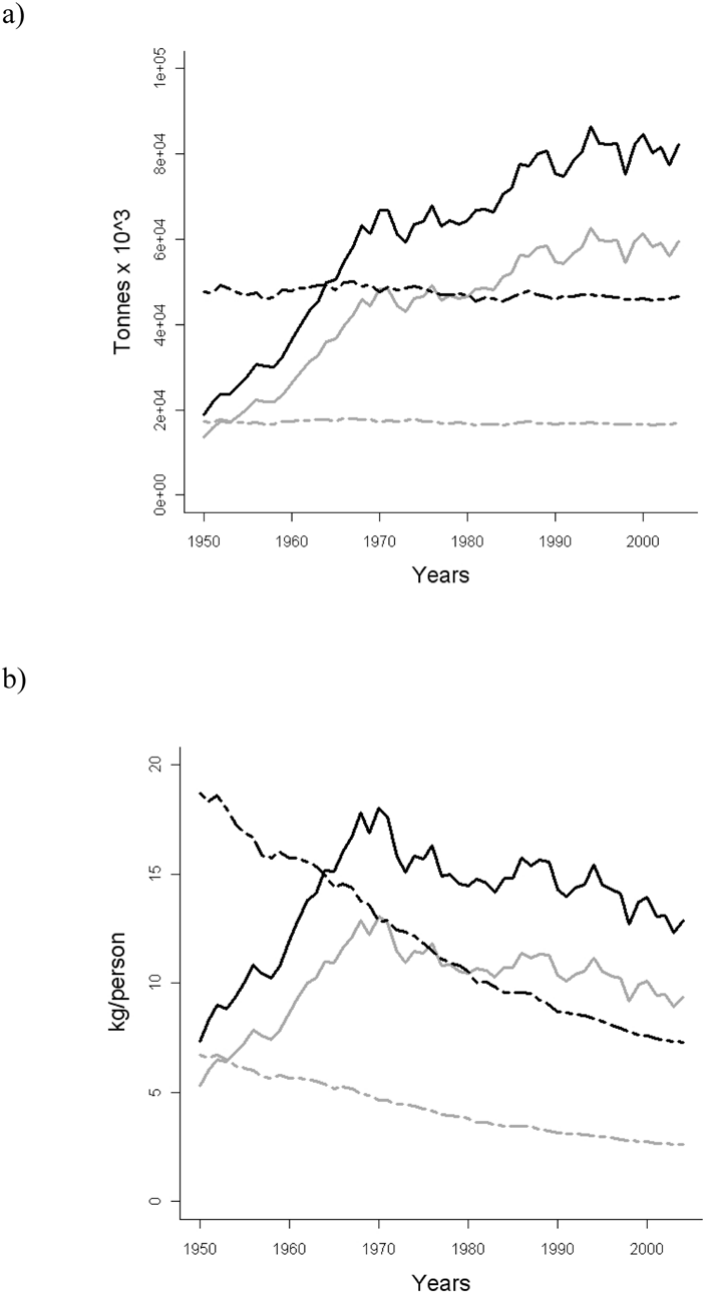
Historical time series (1950–2004) of a. LMEs' catch (t·10^3^·y^−1^) and b. LMEs' catch / total population (kg/person). Catches and total catch per capita take into account official landings (grey) and official landings including discards and 30% unreported estimates (black). Ecosystem-Based Maximum Sustainable catches (total catches and per capita) in LME areas are reported for reference levels p_sust_ = 75% (black dashed) and p_sust_ = 95% (grey dashed).

Total official catches in LMEs, which represent 22% of the global ocean surface but contribute to 75% of global fish catches [16], have continuously increased from the 1950s to the early 1990s, and fluctuated from then on until the present (Figure 4a). The ratio of official catches in LMEs to total human population (catch per capita) also showed an increase from the 1950s to the late 1970s, reaching 13 kg/person at its peak, but declining to 9 kg/person in the 2000s (Figure 4b). Similar patterns emerged when adding discards and IUU estimates to official catches. These patterns reflect the combined effects of an exponential increase in human population and the levelling off of total catches from the 1990s onwards.

Results show that EMSC_75_ were reached in the early 1960s (Figure 4a). Current catches per capita in LMEs may be twice the recommended estimate if 75% probability for sustainability is set as a goal, and 5 times higher if 95% probability is targeted. EMSC estimated for LMEs and FAO areas for 2004 and aggregated on the basis of ecosystem type (Table 1), show that EMSC_75_ may be 47% lower than recorded total catches in temperate and high latitude LMEs, 23% in tropical LMEs, and 62% for upwelling systems if accounting for 30% of IUU. EMSC_95_ estimates are even lower. Conversely, open sea areas show larger EMSC than current catches using conservative values for unreported catches (Table S1 supplementary material).

### Limitations and the way forward

The L index enabled us to quantify the ecosystem overfishing risks at a global scale caused by removing target and non-target species spanning from lower to higher trophic levels by accounting for both bottom-up (primary productivity) and top-down (fishing pressure) effects. However, this analysis could be certainly improved by including higher spatial resolution of catch statistics and new environmental data, such as time series on primary production when such information is available with global coverage. Given the importance of discards and unreported catches on the quantification of ecosystem impacts of fishing, the current assessment using official catch data and conservative IUU% estimates may be too optimistic for several areas known to have high levels of unreported catches and limited monitoring of fisheries such as in open seas and coastal areas under the control of countries with low monitoring capacity [25]–[26]. When we included increasing percentages of IUU in our analyses, the risk of overfishing increased notably. Recreational fisheries' yields are often substantial and their inclusion in future analyses is also recommended [27].

L index results might be affected, other than by uncertainty on input data, by limitations in capturing some dynamic changes occurring in the ecosystem [10]. Indeed, to place the assessment on an absolute scale accounting for historical overfishing would require the determination of pristine ecosystem statuses [4], [28], a complex undertaking. However, the application of the L index to time series data, and time-dynamic modelling [10], allows accounting for past impacts of fishing and regime shifts. Moreover, ecological community responses to fishing pressure and changes in ecosystem production are seldom immediate and delayed effects might be partially accounted when applying L to data time series. Exploitation rates and production levels are likely not homogeneously distributed within LMEs, thus the evaluation presented here might differ from lower-scale approaches. Even so, our results are remarkably consistent with data available from higher-resolution studies [10]. By quantifying the decrease in secondary production, the L index might be considered as indirectly accounting for the risk of extinction and the decrease of species diversity (as biodiversity relates to production [29]), however quantitative evaluations of effects of fishing on diversity in marine ecosystems [e.g. 1]–[2] needs to be done separately. Indeed, given the complex processes involved, ecosystem overfishing evaluations are approximate and the method proposed here represents a framework into which other specific population, community and ecosystem level assessments [e.g.], [ 1]–[2, 5, 8–9] could be folded to account for the exploitation effects at the different hierarchical ecological levels.

Despite some limitations, L index allows for a robust evaluation of the risks of marine ecosystems to overfishing and illustrates when and where fishing became unsustainable at the ecosystem level since the 1950s. It highlights notable risk of ecosystem overfishing for several LME areas, which supply the bulk of marine production to human populations. These results confirm previous concerns about the sustainability of fishing activities at a global scale [5]–[6], [8], [16], [30]. Our results likely imply the need for drastic cuts in total catches, since a redistribution of catches from highly fished LMEs towards open oceans–which, according to our results, might support higher exploitation levels-is not feasible due to ecological, technical, and economic restrictions [2], [30]. Open sea areas have recently registered declines in the sustainability of fishing activities [10, Tables S1, S3, S4, S5], and this situation may actually be worse than indicated by current estimates due to the high levels of IUU catches [25]–[26], Table S2 supplementary material. Notwithstanding the impacts of human exploitation on marine ecosystems were documented as already occurring in early historical times [4], [28], our results show that Humankind has been exploiting marine ecosystems beyond their ability to sustain global catch levels at least from early 1960s. Clearly, fishing is an important factor shaping the ocean and current fishing practices imply a non-negligible risk of ecosystem overfishing, with the subsequent risk of impairing important ecosystem services including the capability to supply food.

## Materials and Methods

### a) The loss in secondary production index

The Loss in secondary production index (L) due to fishing takes into account the amount (quantified by the primary production required, PPR_i_, 8) and the ecological role (summarized here by the trophic level, TL_i_, 5) of caught organisms and incorporates elements of ecosystem function (the primary production at the base of the food web, P_1_, and the average efficiency of energy transfer, TE). As secondary production we intend the production of mass and energy by herbivores and carnivores in the ecosystem.

Thus, L for a given ecosystem can be expressed as a function of the PPR_i_ to sustain catches of each fished species (i = 1, …, m), the Trophic Level (TL_i_) of these species, P_1_, and the TE in the ecosystem's trophic flows:
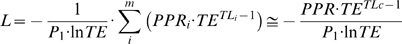
(1)where L can be approximated (rightmost side of Eq. 1) by using the total primary production required to sustain the fishery in the ecosystem (PPR, 8), and the mean trophic level of the catch (TL_c_, 5). Similarly to FiB index and PPR, L weights the catches by using the TL of fished species. However, whereas other indices measure what is taken from the system [9], L addresses the consequences of this removal from the food web [10].

### b) Reference levels for the L index

Reference levels for the L index were quantified by using a set of well documented mass balance models representative of exploited ecosystems distributed worldwide, considering model-independent information on ecosystem status [10]. Values of mean trophic level of the catch (TL_c_), primary production required to sustain the catch (PPR), primary production (P_1_) and transfer efficiency (TE) were calculated from 51 ecosystems using mass-balance model results [31]. Model outputs were used to estimate L indices for each ecosystem following Eq. 1.

Each model was then classified as representing an overfished or sustainably fished ecosystem based on the ecosystem overfishing definition *sensu* Murawski [12]. Ecosystems were considered overfished when cumulative impacts of total catches, non-harvest mortality, and habitat degradation resulted in one or more of the following conditions: (a) Biomasses of species fell below minimum biologically acceptable limits, including the presence of any species threatened with local or biological extinction; (b) Significant decline in diversity of communities or populations as a result of any factor associated with harvesting; (c) Increase of year-to-year variation in populations or catches induced by harvest activities; (d) Decrease in resilience or resistance of the ecosystem to perturbations as a consequence of changes in species demography due to fishing; (e) Cumulative net economic or social benefits lower than would result from alternative fishing patterns or species selection; (f) Fishing mortality impaired the long-term viability of ecologically important, non-resource species. Ecosystems were defined as “sustainably fished” when cumulative impacts of exploitation did not result in any of the above overexploitation symptoms.

Using the frequency distribution of L values it was possible to calculate, for each ecosystem, the probability for the ecosystem to be classified as sustainably fished (p_sust_). For any given value of the L index, say L^*^, the number of models of overexploited ecosystems with L<L^*^, i.e. P(L_1_<L^*^), and the number of models representing sustainably fished ecosystem with L>L^*^, i.e. P(L_2_>L^*^) allowed the estimation of p_sust_ for L^*^ as in the following:
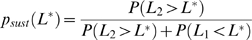
(2)


**Figure 5:**
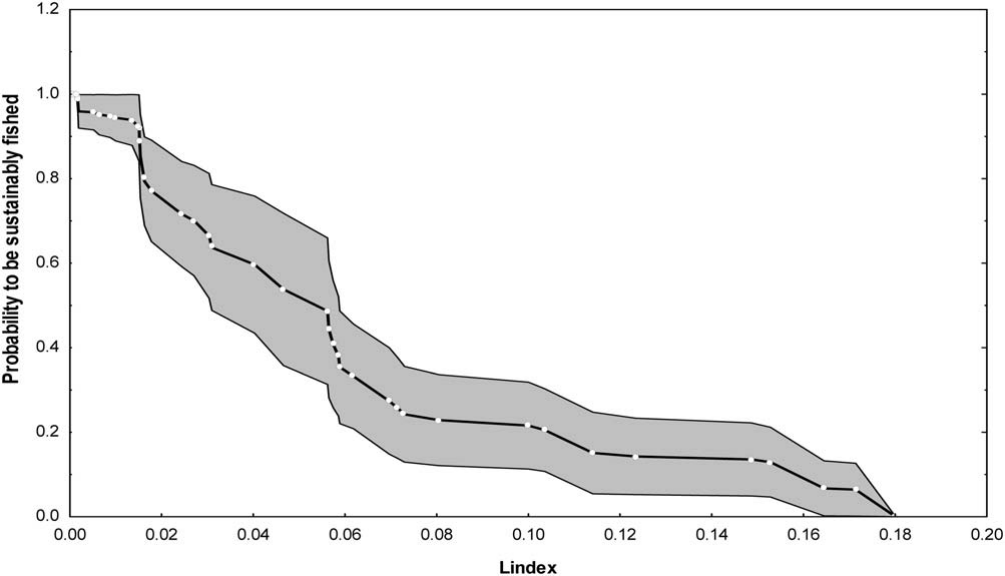
Probability of being sustainably fished (p_sust_) vs Loss in production index (L) values obtained by the application of the analyses of 51 classified models. Nominal values refer to classified models, while averages and confidence intervals are obtained by applying resampling methods (Jackknife; number of random sub-sets, N = 500; length of sub-sets, k = 45). This plot can be used for a) assessing current level of exploitation for an ecosystem from an estimate of L index, b) estimating Ecosystem-based Maximum Sustainable Catches (EMSC), once a p_sust_ value is fixed as a reference level [10]. This figure was modified from Libralato et al. (2008).

A jackknife resampling method [32], which consisted of repeating this non-parametric estimation 500 times with a subset of 45 models randomly chosen, was applied to derive confidence intervals for the identified relationship between L and p_sust_ [10], Figure 5. Such a relationship is based on the hypothesis that, notwithstanding the different fishing pressures and impacts, equally depleted ecosystems show equal values of the L index and thus their sustainability level can be expressed in probability terms. The relationship between L and p_sust_ allows estimating reference values for the L index by fixing any desired reference for sustainability of the fisheries, p_sust_ = p, and estimating the correspondent reference values of the index L = L_p_. By choosing references p_sust_ = 75% and 95%, reference values for the L index were estimated at L_75%_ = 0.021±0.013, L_95%_ = 0.007±0.007 when using primary production as the only source of energy at the base of the food web. Similar references were also estimated using the sum of primary production and detritus flow as the basal production sustaining the ecosystem (P_1_, 10). Limitations of the L index have been discussed in detail elsewhere [10].

### c) Ecosystem-based Maximum Sustainable Catches

The target value L_p_, based on a reference p_sust_ = p, can be achieved with different strategies combining appropriate values of target TL_c_, %PPR, TE and P_1_. Assuming a constant fishing strategy fishery (i.e., TL_c_ constant) and a constant ecosystem function (i.e., TE and P_1_ constant), target %PPR_p_ can be estimated by inverting Eq. (1). On the basis of target and actual PPR (%PPR_p_, %PPR^0^ respectively) along with actual catches (Y^0^), the corresponding Ecosystem-based Maximum Sustainable Catches (EMSC_p_) [11] can be estimated as:
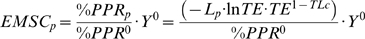
(3)


EMSC_p_ represents the maximum catch that allows to achieve a given reference level of p_sust_ = p. Therefore EMSC_75_ and EMSC_95_ for the two reference values chosen (p_sust_ = 75%, 95%) represent a practical, though approximate, guide for fishery management.

### d) Sensitivity analysis of the L index

In order to explore the propagation of uncertainty of each of the input variables to the value of the L index, local analytical and global numerical sensitivity analyses were performed from representative nominal values (PPR* = 70 gC·m^−2^·y^−1^, P_1_* = 200 gC·m^−2^·y^−1^, TE* = 10% and TLc* = 2.5).

Local sensitivities, calculated as the first order derivative of the L index function [33], represent the change induced in the L index due to changes in each input variable (Figure S1 supplementary material). Results showed that PPR and P_1_ have minor direct influences on the L index, and TE errors have effects of secondary importance, although slightly non-linear, on the L index. Conversely, TLc is the most sensitive input variable in the L index formulation. In particular, a 1% change of PPR, P_1_, TE and TL_c_ results in changes in L in the order of 1%, −1%, 1.9% and 5.8% respectively.

Global sensitivity was also explored by means of MonteCarlo simulations simultaneously accounting for errors in PPR, P1, TL_c_ and TE. In each simulation these parameters are chosen randomly in normal distributions with means equal to the nominal value and realistic standard deviations: a standard error within 10% band was obtained by analysing TE distributions [10]; although uncertainty as high as 100% in PP was found from satellite estimates, operative values of 10% uncertainty appear reasonable for both PP and PPR; finally a 5% standard deviation for TL_c_ seems reasonable given the variability of TL_c_ proposed in the literature. Therefore, 10% standard deviations for PPR, PP and TE and 5% for TL_c_ were considered realistic and were adopted in 10 000 MonteCarlo runs (Figure S2 supplementary material). Results provided an L index distribution with mean: 0.0051, standard deviation: 0.0019, median: 0.0048, 1^st^ and 3^rd^ percentile: 0.0037 and 0.0062 respectively. This deviation is mainly affected by propagation of errors in TLc. However, the robust estimates obtained for L with realistic input parameter uncertainties (L values distributed within an interquartile range of 26% of the median and a standard deviation of 38% of the mean) confirmed that a) L index errors are highly dependent on uncertainty connected with TL_c_, b) TE and other parameters have secondary effects, c) overall, L estimates are robust to realistic levels of uncertainty in input values. These results imply the need for great accuracy when estimating TL_c_, by using the best available estimates for species TLs and catch statistics disaggregated at the lowest possible level.

### e) Data sources

Data concerning landings (t·yr^−1^) from 1950 to 2004, by Large Marine Ecosystems (LMEs) and open sea areas (FAO areas, outside LMEs), were obtained from the Sea Around Us project (SAUP) Global Fisheries Mapping data (version 4.0, http://www.searoundus.org, 13). This database contains the available data from FAO (www.fao.org), complemented with regional and national catch statistics, which are re-expressed on a spatial basis. The SAUP database also provides primary production (mgC·m^−2^·day^−1^) from 1998 and surface (km^2^) estimates for each LME and open sea (FAO area). The primary production data are based on a model whose parameterization varies between biomes and biogeochemical provinces, and that estimates depth-integrated primary production based on chlorophyll pigment concentration as derived from SeaWiFS data and photosynthetically active radiation calculated with a spatial resolution of 9 km [34]–[37]. These data are used to derive estimates of primary production by LMEs, following application of an interpolation procedure, described in www.seaaroundus.org.

The SAUP database was complemented with additional data in order to take into account discards and illegal, unreported or unregulated (IUU) catches for LMEs and open sea (FAO areas). Discard data were available for the period 1992–2002 [14]. For some tropical and temperate and high latitude regions for which such data were lacking, discards were considered as an average proportion of the catches (8% of catches, 14). IUU catches were estimated as 30% of official landings [15]–[17], and a further analysis was performed with additional assumptions of IUU proportion (50%, 100%, 300%, 500% and 1000% of official landings).

Following the climatic distribution [38] and LME definitions [39], catch data were aggregated first by ecosystems, then by ecosystem type, i.e. upwellings, temperate and high latitude regions (including cold-temperate and warm-temperate regions) and tropical regions (including tropical and subtropical regions). Areas with a distribution between temperate and tropical regions were classified within the category most represented in terms of surface. A total of 16 LMEs were located in polar or high-latitude regions. However, they were kept with the temperate systems due to the fact that the transfer efficiency was TE = 14% for areas located in these latitudes, similar to other temperate ecosystems (Libralato et al. 2008).

Trophic level of each caught species or group of species (i), i.e. TL_i_, was taken from Fishbase (www.fishbase.org), the Catalogue of fishes (www.calacademy.org/research/ichthyology) and Cephbase (www.cephbase.utmb.edu) to estimate the mean trophic level (TL_c_) of the catch (Y) [5].
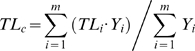
(4)


According to sensitivity analyses, TL_c_ has the major influence on index values. Therefore, this parameter was estimated as accurately as possible for each ecosystem by using disaggregated data. The %PPR was calculated applying the following equation [8]:
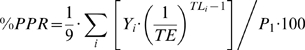
(5)which takes into account transfer efficiency (TE) by ecosystem type and primary production (P_1_) for each area.

Characteristic frequency distribution of TE values by ecosystem type were derived from a set of ecosystem models [10] that were distinguished as belonging to upwelling regions (n = 9; TE: mean = 5.09, sd = 1.47, median = 4, 1^st^ quartile = 3.9, 3^rd^ quartile = 6.1), temperate and high latitude areas (n = 39; TE: mean = 14.25, sd = 5.95, median = 13.7, 1^st^ quartile = 11.3, 3^rd^ quartile = 15.55), tropical areas (n = 21; TE: mean = 10.32, sd = 3.57, median = 9.7, 1^st^ quartile = 7.5, 3^rd^ quartile = 12.9) and global marine ecosystems (n = 91; TE: mean = 11.92, sd = 5.42, median = 11.80, 1^st^ quartile = 8.05, 3^rd^ quartile = 14.3) (TE values reported are in % units). Instead of using a single representative value of TE for each group of models, the original distribution of TE values for each ecosystem type was used for estimating L index, psust and EMSC. This was done by implementing repeated calculations using TE values extracted from the distribution of TE typical for each ecosystem type. This procedure generated distributions for L, psust and EMSC for which statistical indices were calculated (mean, standard deviation, median and interquartile range). Since obtained distributions are seldom symmetrical, the median and interquartile range are reported and used for summarizing results.

Global population estimates were taken from the U.S. Census Bureau, International Data Base (http://www.census.gov/ipc/www/idb/).

## Supporting Information

Figure S1 Variation of L index from nominal value (open circle) as resulting from changes in input parameters (X = TLc, PPR, PP and TE) around their nominal value. Each curve results from sensitivity analyses on one single parameter indicated between parentheses, L(X).
(0.04 MB DOC)

Figure S2 Results of global sensitivity on L index obtained by randomly choosing the four input parameters from normal distributions (μ = nominal values, SD = 10% for all but 5% for TLc). White and red circles represent median and mean values, respectively.
(1.12 MB DOC)

Table S1 Global assessment of ecosystem overfishing for Large Marine Ecosystems (LME) and Open Sea (FAO areas) for the period 2000–2004.
(0.07 MB DOC)

Table S2 Assessment of ecosystem overfishing for Open Sea (FAO areas) for the period 2000–2004 including higher estimates of IUU catches (results follow the ones presented in Table S1).
(0.03 MB DOC)

Table S3 Global assessment of ecosystem overfishing for Large Marine Ecosystems (LME) and Open Sea (FAO areas) for the period 1990–1999.
(0.05 MB DOC)

Table S4 Global assessment of ecosystem overfishing for Large Marine Ecosystems (LME) and Open Sea (FAO areas) for the period 1970–1979.
(0.06 MB DOC)

Table S5 Global assessment of ecosystem overfishing for Large Marine Ecosystems (LME) and Open Sea (FAO areas) for the period 1950–1959.
(0.05 MB DOC)
