# Supplementary material for: Ecosystem Overfishing in the Ocean
Source: PLoS One. 2008 Dec 10;3(12):e3881. doi: 10.1371/journal.pone.0003881 (PMC2587707; doi:10.1371/journal.pone.0003881)
Supplement: Figure S1 — Variation of L index from nominal value (open circle) as resulting from changes in input parameters (X = TLc, PPR, PP and TE) around their nominal value. Each curve results from sensitivity analyses on one single parameter indicated between parentheses, L(X). (0.04 MB DOC) [file pone.0003881.s001.doc]

**Figure S1.**
