# Supplementary material for: Ecosystem Overfishing in the Ocean
Source: PLoS One. 2008 Dec 10;3(12):e3881. doi: 10.1371/journal.pone.0003881 (PMC2587707; doi:10.1371/journal.pone.0003881)
Supplement: Table S2 — Assessment of ecosystem overfishing for Open Sea (FAO areas) for the period 2000–2004 including higher estimates of IUU catches (results follow the ones presented in Table S1). (0.03 MB DOC) [file pone.0003881.s004.doc]

**Table S2.** Assessment of ecosystem overfishing for Open Sea (FAO areas) for the period 2000-2004 including higher estimates of IUU catches (results follow the ones presented in Table S1).
