# Supplementary material for: Ecosystem Overfishing in the Ocean
Source: PLoS One. 2008 Dec 10;3(12):e3881. doi: 10.1371/journal.pone.0003881 (PMC2587707; doi:10.1371/journal.pone.0003881)
Supplement: Table S3 — Global assessment of ecosystem overfishing for Large Marine Ecosystems (LME) and Open Sea (FAO areas) for the period 1990–1999. (0.05 MB DOC) [file pone.0003881.s005.doc]

**Table S3.** Global assessment of ecosystem overfishing for Large Marine Ecosystems (LME) and Open Sea (FAO areas) for the period 1990-1999.
